# Health-related risk behaviors among U.S. childhood cancer survivors: a nationwide estimate

**DOI:** 10.1186/s12885-024-11894-7

**Published:** 2024-02-06

**Authors:** Van T. Nghiem, Jing Jin, Stephen T. Mennemeyer, F. Lennie Wong

**Affiliations:** 1 Department of Health Policy and Organization, University of Alabama at Birmingham School of Public Health, Birmingham, AL USA; 2 Department of Population Sciences, City of Hope, Duarte, CA USA

**Keywords:** Childhood cancer survivors, Risk behaviors, Long-term follow-up, Lifestyle, Behavioral medicine

## Abstract

**Background:**

Childhood cancer survivors (CCS) are subject to a substantial burden of treatment-related morbidity. Engaging in health protective behaviors and eliminating risk behaviors are critical to preventing chronic diseases and premature deaths. This study is aimed to provide updated information on currently smoking, physical inactivity, binge drinking patterns and associated factors among CCS using a nationwide dataset.

**Methods:**

We constructed a sample of CCS (cancer diagnosis at ages < 21y) and healthy controls (matched on age, sex, residency, race/ethnicity) using 2020 Behavioral Risk Factor Surveillance System. We used Chi-square tests and Wilcoxon rank-sum test to examine differences in sociodemographics and clinical characteristics between two groups. Logistic, ordinal regression and multivariable models (conditional models for matching) were used to determine factors associated with risk behaviors.

**Results:**

The final sample (18-80y) included 372 CCS and 1107 controls. Compared to controls, CCS had a similar proportion of binge drinking (~ 18%) but higher prevalence of currently smoking (26.6% vs. 14.4%, *p* < 0.001), physical inactivity (23.7% vs. 17.7%, *p* = 0.012), and of having 2-or-3 risk behaviors (17.2% vs. 8.1%, *p* < 0.001). Younger age, lower educational attainment, and having multiple chronic health conditions were associated with engaging in more risk behaviors among CCS. Females, compared to male counterparts, had lower odds of binge drinking (adjusted odds ratio (aOR) = 0.30, 95% confidence interval (CI): 0.16–0.57) among CCS but not in all sample. Having multiple chronic health conditions increased odds of both currently smoking (aOR = 3.52 95%CI: 1.76–7.02) and binge drinking (aOR = 2.13 95%CI: 1.11–4.08) among CCS while it only increased odds of currently smoking in all sample.

**Discussion:**

Our study provided risk behavior information for wide age-range CCS, which is currently lacking. Every one in four CCS was currently smoking. Interventions targeting risk behavior reduction should focus on CCS with multiple chronic health conditions.

**Supplementary Information:**

The online version contains supplementary material available at 10.1186/s12885-024-11894-7.

## Introduction

Thanks to advances in cancer care, there has been significant improvement in life expectancy for childhood cancer survivors, resulting in a growing population of them currently estimated to be over 500,000 in the United States (US) [1]. However, childhood cancer survivors carry a substantial burden of treatment-related morbidity such as subsequent malignancies, cardiovascular disease, and other adverse health outcomes [2]. Engaging in health protective behaviors and eliminating unhealthy behaviors are critical to prevent chronic diseases and premature death [3–5].

Previous reports have described health risk behaviors in childhood cancer survivors. In 2002, the Childhood Cancer Survivors Study (CCSS) used a hospital-based cohort of survivors with ≥ 5 years of survival and found the prevalence of smoking among childhood cancer survivors between the ages of 18 and 49 years at 17%, which was lower than the prevalence observed in the general population [6]. A 2007 report from the CCSS cohort found that childhood acute lymphoblastic leukemia survivors were less likely to report non-leisure physical activity compared to controls [7]. These and a few other articles reporting risk behaviors among U.S. childhood cancer survivors date back to the 2010s or earlier and have primarily utilized the CCSS cohort – a hospital-based resource [6–9]. This hospital-based data recruited participants in clinical settings, i.e., through clinics and medical centers. This recruitment approach for the CCSS cohort in clinical settings is different from the recruitment approach in the community setting (i.e., population-based phone surveys). This recruitment approach in clinical settings at times could demonstrate challenges, e.g., barriers in recruiting minorities participants [10]. and limited geographics [11].

Updated information on these risk behaviors among U.S. childhood cancer survivors is essential to provide timely public health interventions. Using the Los Angeles—Surveillance, Epidemiology, and End Results Program data (1996 – 2010), Ng et al*.*studied substance misuse among young adult childhood cancer survivors (median age: 26.5y). They found that, among these survivors, substance misuse behavior proportion was 34% for binge drinking, 11% for cigarette use, and 7% for e-cigarette/vaporizer use [12]. Cappelli et al*.*found that, between two timepoints of data collection (from 2007 – 2009 to 2015 – 2018), rates of 30-day use increased for binge drinking (from 26 to 38%), and cigarette tobacco (from 9 to 12%) among a sample of 127 young adult childhood cancer survivors recruited for a project in Los Angeles [13]. Generally, more recent information on health-related risk behaviors among childhood cancer survivors is scarce. Also, there is paucity of risk behavior information on older survivors of childhood cancer [14, 15]. Thus it is important to study risk behaviors among U.S. childhood cancer survivors spanning across a wider adult age range, using datasets with the potential of greater generalizability.

In our study, we used the Behavioral Risk Factor Surveillance System (BRFSS), a nationally representative dataset, to provide updated information on lifestyle health-related risk behaviors (i.e., currently smoking, binge drinking, and physical inactivity) among U.S. childhood cancer survivors. Our findings will be helpful for the development of health programs aiming at promoting healthy lifestyle and ultimately to improve health outcomes and quality of life for U.S. childhood cancer survivors.

## Methods

### Study design and population

Data for our study came from the 2020 BRFSS. This is an annual telephone-based (both landline and cellphone) survey that captures sociodemographics, health-related risk factors, chronic health conditions, and use of preventive services among U.S. non-institutionalized residents across 50 states, the District of Columbia and several territories. The survey includes a core component soliciting information on demographic characteristics, current health behaviors (e.g., smoking, seatbelt use) and other information from all participating states. States are also offered optional modules (e.g., adverse childhood experience, cancer survivorship, healthcare access, lung cancer screening)  [16]. In our study, childhood cancer survivors were identified as survey participants who ever had cancer at age 20y or younger. Healthy controls were selected from the pool of survey participants who did not have cancer at age of ≤ 20y based on an exact match (1:3) [17, 18]. on age, sex, race/ethnicity and state of residency. Because we used exact matching, we did not use the sample weights in the BRFSS. Inverse weighting is used in population-based case–control studies that use a complex sampling scheme for ascertaining cases and controls because the distribution of the confounding variables may be different between cases and controls [19]. In our study, matching ensured that the distribution of the potential confounding variables was similar between cases and controls. Hence weighting is not needed and irrelevant.

### Outcomes and covariates

Our study examined three risk behaviors – currently smoking, physical inactivity and binge drinking. Currently smoking participants were selected by satisfying both criteria: 1) those who reported to have smoked at least 100 cigarettes in their entire life, and 2) those who were everyday/someday smokers at the time of the survey. Physical inactivity used a yes/no response to the question on physical activity or exercise during the past 30 days other than their regular job. Binge drinking was defined as having five-or-more drinks (for males) or having four-or-more drinks (for females) on one occasion. We also constructed a composite outcome measure, which was the three-level categories of the count of risk behaviors: 0 (no risk behavior), 1 (1 risk behavior) and 2 (2 or 3 risk behaviors).

We selected covariates for the assessments of correlates with the three risk behaviors based on previous studies [20–22]. These covariates included clinical and sociodemographic variables, and variables of perceived vulnerability and health concerns.

### Statistical methods

Chi-squared tests and the Wilcoxon rank sum test were used to provide descriptive statistics on the prevalence of risk behaviors and participant characteristics. Univariate, ordinal and multivariable logistic regression models were used for assessments of correlates with these behaviors and the count of risk behaviors. Accounting for matching between childhood cancer survivors and controls, we used conditional models for assessments. Selection of the covariates for the multivariable models was based on the criteria of *p* < 0.20 in the univariate analyses as well as covariates that were identified in previous studies [20–25]. We did not include highly correlated covariates simultaneously in multivariable models (i.e., age and length of follow-up – time elapsing from diagnosis of the primary cancer, among childhood cancer survivors, education and income) [26]. All statistical tests were two-sided at the significance level of 0.05. We conducted all analyses on SAS® 9.4 software (SAS® Institute Inc., Cary, NC). We followed the Strengthening the Reporting of Observational Studies in Epidemiology (STROBE) Statement: Guidelines for Reporting Observational Studies to report findings from our study [27].

We used data from the publicly available BRFSS that had no individually identifiable information and was not deemed human subjects research. Consequently, our study was exempt from further consideration under institutional review boards.

## Results

### Sample characteristics

Our final sample included 372 childhood cancer survivors and 1,107 matched healthy controls (Supplemental material). The survivors completed the survey at a median age of 45.5y (range 18-80y). The majority were White (82.3%) and female (61.1%). Childhood cancer survivors were less likely to be employed (54.0% vs. 60.7%) and had a higher proportion reporting to be unable to work (11.3% vs. 4.3%, *p* < 0.001) than controls. Childhood cancer survivors reported lower annual household income (*p* = 0.021). A higher proportion of childhood cancer survivors were divorced/ widowed/ separated when compared to controls (27.4% vs. 20.4%, *p* = 0.015; Table 1).

**Table 1:** Characteristics of childhood cancer survivors and controls

Characteristics	CCS (*n* = 372)		Controls (*n* = 1107)		*p*-value
	**N**	**%**	**N**	**%**	
**Demographic and socioeconomic factors**					
Age at survey (years)					
18 – 39	154	41.4	459	41.5	NA
40 – 64	133	35.8	394	35.6	
65 and older	85	22.8	254	22.9	
Mean (min–max) [standard deviation]	47.6 (18–80) [18.0]		47.6 (18–80) [18.0]		
Sex					
Male	145	39.0	431	38.9	NA
Female	227	61.0	676	61.1	
Race/ ethnicity					
White only, Non-Hispanic	306	82.3	918	82.9	NA
Black only, Non-Hispanic	14	3.8	42	3.8	
Others	52	14.0	147	13.3	
Region					
Northeast	84	22.6	251	22.7	NA
Midwest	78	21.0	232	21.0	
South	88	23.7	259	23.4	
West	122	32.8	365	33.0	
Education Level					
High school and below	115	30.9	303	27.4	0.400
Attended college or technical school	105	28.2	319	28.8	
College or technical school and above	152	40.9	485	43.8	
Employment					
Employed/self-employed	201	54.0	672	60.7	< .001***
Out of work/A homemaker/A student	58	15.6	180	16.3	
Retired	71	19.1	207	18.7	
Unable to work	42	11.3	48	4.3	
Marital status					
Married/A member of an unmarried couple	186	50.0	627	56.6	0.015*
Divorced/widowed/separated	102	27.4	226	20.4	
Never married	84	22.6	254	22.9	
Number of children in household					
No children	245	65.9	693	62.6	0.259
One or more	127	34.1	414	37.4	
Any healthcare coverage					
Yes	338	90.9	1007	91.0	0.951
No	34	9.1	100	9.0	
Annual household income					
Less than $15,000	45	12.1	75	6.8	0.021*
$15,000 to less than $25,000	49	13.2	133	12.0	
$25,000 to less than $35,000	36	9.7	111	10.0	
$35,000 to less than $50,000	43	11.6	145	13.1	
$50,000 or more	199	53.5	643	58.1	
**Clinical factors**					
Days with poor mental health in past 30 days					
Zero day	161	43.3	640	57.8	< .001***
At least 1 day	211	56.7	467	42.2	
Days with poor physical health in past 30 days					
Zero day	202	54.3	778	70.3	< .001***
At least 1 day	170	45.7	329	29.7	
Obesity					
Yes	235	63.2	696	62.9	0.918
No	137	36.8	411	37.1	
Number of chronic health conditions					
None or 1	195	52.4	880	79.5	< .001
> 1	177	47.6	227	20.5	

*CCS *Childhood cancer survivors, *NA *Not applicable *p*-values because no tests were executed on the covariates that were used for the matching step

^*^p < 0.05, **p < 0.01, ***p < 0.001

Childhood cancer survivors, compared to controls, were more likely to report at least 1 day with poor mental health (56.7% vs. 42.2%, *p* < 0.001) or at least 1 day with poor physical health (45.7% vs. 29.7%, *p* < 0.001) within the last 30 days. While the prevalence of obesity was comparable for the survivors and controls, childhood cancer survivors were two times as likely to report multiple chronic health conditions (47.5% vs. 20.5%, *p* < 0.001, Table 1). Childhood cancer survivors had statistically significantly higher prevalence of the following chronic health conditions: asthma, diabetes, arthritis, depressive disorder, heart attack, stroke, coronary heart disease, chronic obstructive pulmonary disease and kidney disease (data not shown in tables). Mean follow-up since childhood cancer diagnosis for childhood cancer survivors was 33y (standard deviation: 18.7y), median follow-up was 32y (data not shown in tables.)

### Risk behaviors among childhood cancer survivors vs. healthy controls

**Fig. 1:**
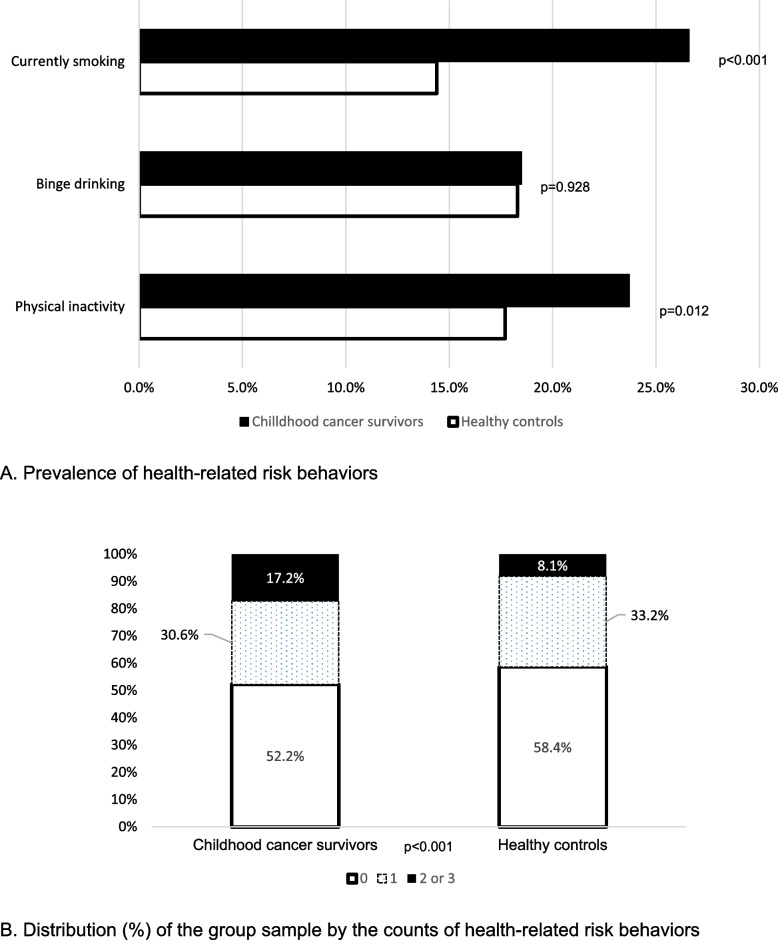
Comparisons of prevalence of lifestyle health-related risk factors among childhood cancer survivors and controls (2020). Childhood cancer survivors had a higher prevalence of currently smoking (26.6% vs. 14.4%) and of physical inactivity (23.7% vs. 17.7%) than controls. Childhood cancer survivors and controls had comparable prevalence of binge drinking, 18.3% and 18.5%, respectively. **A** Prevalence of health-related risk behaviors. **B** Distribution of the count of lifestyle health-related risk behaviors

Childhood cancer survivors were significantly more likely to be current smokers (26.6% vs. 14.4%, *p* < 0.001) compared to controls. Twenty-four percent (23.7%) of childhood cancer survivors were physically inactive compared to 17.7% in controls (*p* = 0.012). The prevalence of binge drinking among childhood cancer survivors and controls was comparable (~ 18%, *p* = 0.928). Childhood cancer survivors had a higher proportion of > 1 risk behavior (17.2% vs. 8.4%, *p* < 0.001, Fig. 1).

In the multivariable models, childhood cancer survivors had 21% higher odds of being a current smoker than controls, 22% higher odds of physical inactivity; however, these differences were not statistically significant. Having > 1 chronic health condition was a statistically significant factor associated with higher odds of currently smoking and and higher odds of physical inactivity in the multivariable regression models. Meanwhile, higher educational attainment was a statistically significant factor associated with lower odds of both currently smoking and physical inactivity. Divorced/widowed/separated participants had threefold odds of currently smoking compared to participants that were married or members of an unmarried couple. Also, participants reporting at least 1 day of poor physical health in the past 30 days had 1.55-fold odds of physical inactivity. Obese participants had two-fold odds of physical inactivity compared to non-obese counterparts. There was no multivariable analysis for binge drinking behavior since only one covariate, i.e., reporting ≥ 1 day with poor mental health in the past 30 days was associated with binge drinking as well as satisfied the variable selection criteria from the univariate analysis (Table 2). Childhood cancer survivors were at a 1.33-fold odds of having ≥ 2 risk behaviors, but this increased odds was not statistically significant in the multivariable model (Table 3).

**Table 2:** Factors associated with smoking, physical inactivity and binge drinking in childhood cancer survivors vs. controls

Characteristics	Currently smoking				Physical inactivity				Binge drinking	
	**Univariate**		**Multivariable**		**Univariate**		**Multivariable**		**Univariate**	
	**OR (95% CI)**	***p*-value**	**aOR (95% CI)**	***p*-value**	**OR (95% CI)**	***p*-value**	**aOR (95% CI)**	***p*-value**	**OR (95% CI)**	***p*-value**
**Demographic and socioeconomic factors**										
Group (CCS vs. Control)	2.19 (1.64, 2.93)	< .001***	1.21 (0.82, 1.79)	0.325	1.47 (1.10, 1.97)	0.009**	1.22 (0.85, 1.75)	0.270	1.02 (0.74, 1.39)	0.926
Education Level										
High school and below	Ref	< .001***	Ref	< .001***	Ref	< .001***	Ref	< .001***	Ref	0.324
Attended college or technical School	0.43 (0.28, 0.64)		0.49 (0.31, 0.79)		0.72 (0.49, 1.06)		0.96 (0.62, 1.49)		1.34 (0.89, 2.01)	
College or technical school and above	0.15 (0.10, 0.23)		0.26 (0.15, 0.43)		0.21 (0.14, 0.32)		0.36 (0.22, 0.59)		1.26 (0.86, 1.84)	
Employment										
Employed/self-employed	Ref	< .001***	HC		Ref	< .001***	HC		Ref	0.239
Out of work/A homemaker/A student	1.32 (0.85, 2.05)				1.26(0.79, 2.00)				1.03 (0.66, 1.60)	
Retired	0.70 (0.33, 1.51)				0.81 (0.43, 1.50)				0.48 (0.18, 1.29)	
Unable to work	3.91 (2.18, 7.02)				3.55 (2.00, 6.32)				0.57 (0.27, 1.19)	
Marital status										
Married/A member of an unmarried couple	Ref	< .001***	Ref	< .001***	Ref	0.034*	Ref	0.925	Ref	0.634
Divorced/widowed/separated	4.14 (2.74, 6.25)		3.04 (1.82, 5.06)		1.65 (1.13, 2.40)		0.92 (0.59, 1.45)		1.23 (0.79, 1.90)	
Never married	2.00 (1.28, 3.13)		1.66 (0.96, 2.88)		1.21 (0.74, 1.97)		0.93 (0.53, 1.61)		0.99 (0.65, 1.51)	
Number of Children in household: (Ref. to No children)										
One or more	1.01 (0.70, 1.47)	0.962	NA		0.99 (0.66, 1.48)	0.945	NA		0.87 (0.60, 1.26)	0.471
Any healthcare coverage (Ref. to No)										
Yes	0.55 (0.34, 0.89)	0.016*	1.23 (0.66, 2.31)	0.518	0.53 (0.30, 0.91)	0.021*	0.67 (0.36, 1.26)	0.211	1.02 (0.62, 1.67)	0.950
Annual household income										
Less than $15,000	Ref	< .001***	Ref	0.018*	Ref	< .001***	Ref	< .001***	Ref	0.265
$15,000 to less than $25,000	0.56 (0.29, 1.06)		0.92 (0.44, 1.93)		1.53 (0.79, 2.94)		2.01 (0.99, 4.09)		0.96 (0.45, 2.02)	
$25,000 to less than $35,000	0.44 (0.22, 0.88)		0.76 (0.34, 1.71)		1.05 (0.54, 2.04)		1.53 (0.73, 3.19)		0.80 (0.37, 1.76)	
$35,000 to less than $50,000	0.19 (0.09, 0.38)		0.50 (0.22, 1.11)		0.56 (0.29, 1.11)		0.94 (0.45, 1.97)		0.96 (0.45, 2.05)	
$50,000 or more	0.11 (0.06, 0.19)		0.38 (0.19, 0.77)		0.28 (0.16, 0.49)		0.62 (0.32, 1.22)		1.35 (0.71, 2.54)	
**Clinical factors**										
Days with poor mental health in past 30 days (Ref. to zero day)										
At least 1 day	1.99 (1.43, 2.77)	< .001***	1.19 (0.77, 1.83)	0.427	1.71 (1.23, 2.39)	0.001**	1.20 (0.81, 1.80)	0.363	1.74 (1.24, 2.45)	0.001**
Days with poor physical health in past 30 days (Ref. to zero day)										
At least 1 day	2.37 (1.70, 3.31)	< .001***	1.41 (0.91, 2.19)	0.122	2.09 (1.50, 2.92)	< .001***	1.55 (1.04, 2.32)	0.033*	1.04 (0.74, 1.45)	0.840
Obesity (Ref. to No)										
Yes	0.95 (0.69, 1.30)	0.745	NA		2.09 (1.48, 2.96)	< .001***	1.96 (1.33, 2.86)	< .001***	1.12 (0.81, 1.56)	0.495
Number of chronic health conditions (Ref. to none or 1)										
> 1	4.81 (3.25, 7.11)	< .001***	2.47 (1.45, 4.22)	0.001**	2.77 (1.96, 3.91)	< .001***	1.56 (1.01, 2.40)	0.044*	1.12 (0.78, 1.63)	0.537

*CCS *Childhood cancer survivor, *HC *Highly correlated with one of the covariates included in the multivariable models, *NA *Not applicable covariate for not satisfying the variable selection criterium in the model fitting step

^*^p < 0.05, **p < 0.01, ***p < 0.001

**Table 3:** Factors associated with the count of health-related risk factors in childhood cancer survivors vs. controls

Demographics	Risk behaviors (*N* = 1322)				Risk behaviors (*N* = 1479)				Risk behaviors (*N* = 1479)			
	**1 risk behavior vs. none**				** ≥ 1 risk behaviors vs. none**				** ≥ 2 risk behaviors vs. ≤ 1**			
	**Univariate**		**Multivariable**		**Univariate**		**Multivariable**		**Univariate**		**Multivariable**	
	**OR (95% CI)**	***p*-value**	**aOR (95% CI)**	*p*-value	**OR (95% CI)**	***p-*value**	**aOR (95% CI)**	***p*-value**	**OR (95% CI)**	***p*-value**	**aOR (95% CI)**	***p*-value**
**Demographic and socioeconomic factors**												
Group (CCS vs. Control)	1.08 (0.82, 1.41)	0.607	0.89 (0.66, 1.21)	0.465	1.30 (1.03, 1.66)	0.030	0.97 (0.73, 1.29)	0.850	2.35 (1.65, 3.35)	< 0.001	1.33 (0.85, 2.09)	0.216
Education Level												
High school and below	Ref	< 0.001 ***	Ref	0.004**	Ref	< 0.001 ***	Ref	< 0.001 ***	Ref	< 0.001 ***	Ref	0.007 **
Attended college or technical school	0.73 (0.51, 1.05)		0.84 (0.57, 1.24)		0.70 (0.51, 0.97)		0.84 (0.59, 1.19)		0.64 (0.40, 1.05)		0.75 (0.43, 1.30)	
College or technical school and above	0.38 (0.27, 0.53)		0.53 (0.36, 0.78)		0.32 (0.24, 0.44)		0.47 (0.33, 0.67)		0.22 (0.13, 0.38)		0.37 (0.20, 0.69)	
Employment												
Employed/self-employed	Ref	0.020*	NA		Ref	< 0.001 ***	NA		Ref	< 0.001 ***	NA	
Out of work/A homemaker/A student	1.40 (0.92, 2.11)				1.31 (0.91, 1.88)				0.98 (0.56, 1.71)			
Retired	0.80 (0.46, 1.38)				0.71 (0.42, 1.21)				0.33 (0.09, 1.15)			
Unable to work	2.28 (1.19, 4.37)				2.91 (1.67, 5.08)				3.66 (1.80, 7.44)			
Marital status												
Married/A member of an unmarried couple	Ref	< 0.001 ***	Ref	0.017*	Ref	< 0.001 ***	Ref	0.004 **	Ref	< 0.001 ***	Ref	0.120
Divorced/widowed/separated	2.37 (1.65, 3.41)		1.73 (1.16, 2.59)		2.57 (1.85, 3.55)		1.85 (1.28, 2.66)		2.62 (1.57, 4.36)		1.92 (1.02, 3.64)	
Never married	1.22 (0.82, 1.83)		0.90 (0.58, 1.41)		1.37 (0.96, 1.96)		1.05 (0.70, 1.56)		1.46 (0.87, 2.47)		1.33 (0.72, 2.48)	
Number of children in household (Ref. to No children)												
One or more	0.66 (0.46, 0.94)	0.023*	0.69 (0.46, 1.02)	0.062	0.78 (0.57, 1.06)	0.111	0.80 (0.56, 1.13)	0.200	1.29 (0.81, 2.05)	0.280	NA	
Any healthcare coverage (Ref. to No)												
Yes	0.54 (0.33, 0.87)	0.011*	0.82 (0.49, 1.39)	0.459	0.56 (0.36, 0.87)	0.010*	0.85 (0.52, 1.39)	0.529	0.72 (0.39, 1.33)	0.289	NA	
Annual household income												
Less than $15,000	Ref	< 0.001 ***	Ref	0.044 *	Ref	< 0.001 ***	Ref	0.011 *	Ref	< 0.001 ***	Ref	0.720
$15,000 to less than $25,000	1.05 (0.56, 1.98)		1.31 (0.67, 2.55)		0.89 (0.51, 1.57)		1.08 (0.59, 1.96)		0.69 (0.31, 1.50)		1.13 (0.45, 2.82)	
$25,000 to less than $35,000	0.67 (0.35, 1.29)		0.93 (0.46, 1.86)		0.54 (0.30, 0.98)		0.76 (0.40, 1.43)		0.50 (0.21, 1.18)		0.90 (0.34, 2.38)	
$35,000 to less than $50,000	0.51 (0.27, 0.96)		0.79 (0.40, 1.55)		0.39 (0.22, 0.69)		0.65 (0.35, 1.20)		0.24 (0.10, 0.58)		0.63 (0.23, 1.74)	
$50,000 or more	0.31 (0.18, 0.53)		0.61 (0.33, 1.12)		0.23 (0.14, 0.38)		0.51 (0.29, 0.88)		0.22 (0.11, 0.43)		0.78 (0.32, 1.87)	
**Clinical factors**												
Days with poor mental health in past 30 days (Ref. to zero day)												
At least 1 day	1.50 (1.13, 2.00)	0.006 **	1.33 (0.97, 1.83)	0.083	1.85 (1.42, 2.39)	< 0.001 ***	1.52 (1.13, 2.03)	0.006 **	2.70 (1.75, 4.16)	< 0.001 ***	1.45 (0.86, 2.45)	0.166
Days with poor physical health in past 30 days (Ref. to zero day)												
At least 1 day	1.80 (1.34, 2.41)	< .001 ***	1.53 (1.10, 2.12)	0.011 *	1.98 (1.52, 2.59)	< 0.001 ***	1.43 (1.06, 1.95)	0.020 *	2.06 (1.35, 3.14)	< 0.001 ***	1.03 (0.60, 1.75)	0.924
Obesity (Ref. to No)												
Yes	1.21 (0.92, 1.60)	0.180	0.94 (0.69, 1.28)	0.692	1.31 (1.02, 1.69)	0.038 *	0.88 (0.67, 1.17)	0.381	1.39 (0.92, 2.11)	0.117	1.19 (0.73, 1.95)	0.481
Number of chronic health conditions (Ref. to none or 1)												
> 1	1.78 (1.30, 2.45)	< .001 ***	1.15 (0.79, 1.68)	0.452	2.38 (1.80, 3.16)	< .001 ***	1.39 (0.99, 1.95)	0.059	5.26 (3.25, 8.51)	< .001 ***	2.81 (1.51, 5.20)	0.001 **

*CCS* Childhood cancer survivors, *NA* Not applicable covariate for not satisfying the variable selection criterium in the model fitting step

^*^p < 0.05, **p < 0.01, ***p < 0.001

### Factors associated with health-related risk behaviors among childhood cancer survivors

#### Currently smoking

The oldest childhood cancer survivors (65y +) had much lower odds of currently smoking (adjusted odds ratio (aOR) [95% confidence interval (CI)] = 0.17 [0.06–0.52]) compared to the youngest counterparts (18-38y). Compared to those with high school degree or below, survivors attending college or technical school and survivors finishing college or technical school and above had lower odds of currently smoking (aOR [95%CI] = 0.42 [0.20–0.86]; aOR [95%CI] = 0.25 [0.25–0.54]; respectively). Having > 1 chronic health condition was associated with higher odds of currently smoking (aOR [95%CI] = 3.52 [1.76–8.02], Table 4).

**Table 4:** Factors associated with currently smoking, physical inactivity and binge drinking among childhood cancer survivors

Characteristics	Currently smoking				Physical inactivity				Binge drinking			
	**Univariate**		**Multivariable**		**Univariate**		**Multivariable**		**Univariate**		**Multivariable**	
	**Odds Ratio** **(95% CI)**	***p*-value**	**Odds Ratio** **(95% CI)**	***p*-value**	**Odds Ratio** **(95% CI)**	***p*-value**	**Odds Ratio** **(95% CI)**	***p*-value**	**Odds Ratio** **(95% CI)**	***p*-value**	**Odds Ratio** **(95% CI)**	***p*-value**
**Demographic and socioeconomic factors**												
Age at survey (years)												
18–39	Ref	< 0.001 ***	Ref	0.008 **	Ref	0.508	Ref	0.165	Ref	< 0.001 ***	Ref	0.003 **
40–64	0.71 (0.43, 1.18)		0.61 (0.31, 1.19)		1.36 (0.79, 2.34)		1.77 (0.96, 3.26)		0.36 (0.20, 0.65)		0.42 (0.21, 0.85)	
65 and older	0.19 (0.09, 0.43)		0.17 (0.06, 0.52)		1.06 (0.56, 2.00)		1.16 (0.54, 2.46)		0.11 (0.04, 0.33)		0.15 (0.05, 0.52)	
Sex (Ref. to Male)												
Female	1.90 (1.15, 3.13)	0.012 *	1.19 (0.61, 2.29)	0.612	0.90 (0.55, 1.47)	0.671	0.65 (0.37, 1.16)	0.146	0.41 (0.24, 0.70)	0.001 **	0.30 (0.16, 0.57)	< 0.001 ***
Region												
Northeast	Ref	0.692	Ref	0.937	Ref	0.007 **	Ref	0.108	Ref	0.929	Ref	0.947
Midwest	1.18 (0.59, 2.37)		0.80 (0.32, 1.99)		2.15 (0.99, 4.63)		1.82 (0.78, 4.21)		1.19 (0.53, 2.66)		0.95 (0.38, 2.35)	
South	1.33 (0.68, 2.60)		1.04 (0.43, 2.52)		2.97 (1.42, 6.20)		2.27 (1.03, 5.03)		1.29 (0.59, 2.79)		1.13 (0.47, 2.74)	
West	0.94 (0.49, 1.79)		0.98 (0.43, 2.21)		1.20 (0.57, 2.54)		1.11 (0.50, 2.47)		1.10 (0.53, 2.30)		0.89 (0.38, 2.08)	
Race												
White only, Non-Hispanic	Ref	0.113	Ref	0.734	Ref	0.209	Ref	0.232	Ref	0.115	Ref	0.479
Black only, Non-Hispanic	1.23 (0.38, 4.04)		0.58 (0.12, 2.77)		1.71 (0.56, 5.26)		1.31 (0.37, 4.63)		1.36 (0.37, 5.06)		0.92 (0.20, 4.33)	
Others	1.93 (1.04, 3.57)		1.13 (0.52, 2.47)		0.56 (0.25, 1.24)		0.48 (0.20, 1.16)		2.03 (1.04, 3.97)		1.60 (0.74, 3.45)	
Education Level												
High school and below	Ref	< .001 ***	Ref	0.002 **	Ref	0.003 **	Ref	0.242	Ref	0.002 **	Ref	0.056
Attended college or technical School	0.45 (0.25, 0.78)		0.42 (0.20, 0.86)		0.96 (0.54, 1.71)		1.04 (0.54, 2.00)		0.65 (0.35, 1.22)		0.81 (0.39, 1.65)	
College or technical school and above	0.15 (0.08, 0.27)		0.25 (0.12, 0.54)		0.39 (0.21, 0.71)		0.60 (0.29, 1.22)		0.31 (0.16, 0.59)		0.41 (0.19, 0.86)	
Employment												
Employed/self-employed	Ref	< 0.001 ***	NA		Ref	0.010 *	NA		Ref	0.021 *	NA	
Out of work/A homemaker/A student	1.55 (0.83, 2.90)				1.13 (0.56, 2.28)				1.14 (0.57, 2.26)			
Retired	0.22 (0.09, 0.58)				1.05 (0.54, 2.03)				0.16 (0.05, 0.52)			
Unable to work	3.56 (1.79, 7.07)				3.22 (1.60, 6.48)				0.84 (0.36, 1.94)			
Marital status												
Married/A member of an unmarried couple	Ref	0.009 **	Ref	0.385	Ref	0.278	NA		Ref	0.029 *	Ref	0.175
Divorced/widowed/separated	2.29 (1.33, 3.94)		1.57 (0.73, 3.40)		1.52 (0.88, 2.64)				0.50 (0.24, 1.04)		0.45 (0.19, 1.05)	
Never married	1.71 (0.95, 3.08)		0.86 (0.38, 1.93)		1.00 (0.53, 1.87)				1.48 (0.81, 2.71)		0.79 (0.38, 1.65)	
Number of Children in household: (Ref. to No children)												
One or more	2.60 (1.62, 4.17)	< 0.001 ***	2.30 (1.22, 4.31)	0.010 *	0.81 (0.49, 1.36)	0.434	NA		1.63 (0.96, 2.79)	0.071	0.97 (0.52, 1.81)	0.916
Any healthcare coverage (Ref. to No)												
Yes	0.37 (0.18, 0.75)	0.006 **	0.72 (0.28, 1.88)	0.506	0.85 (0.38, 1.89)	0.686	NA		0.51 (0.23, 1.12)	0.092	0.82 (0.32, 2.09)	0.681
Annual household income												
Less than $15,000	Ref	< 0.001 ***	Ref	0.062	Ref	0.002 **	Ref	0.032 *	Ref	0.556	NA	
$15,000 to less than $25,000	0.38 (0.17, 0.88)		0.50 (0.18, 1.41)		0.80 (0.33, 1.92)		1.19 (0.45, 3.12)		0.43 (0.15, 1.29)			
$25,000 to less than $35,000	0.21 (0.08, 0.55)		0.35 (0.10, 1.18)		1.60 (0.65, 3.95)		2.80 (0.98, 8.01)		0.62 (0.20, 1.87)			
$35,000 to less than $50,000	0.15 (0.06, 0.38)		0.23 (0.07, 0.76)		0.69 (0.27, 1.73)		1.39 (0.48, 4.05)		0.94 (0.35, 2.50)			
$50,000 or more	0.10 (0.05, 0.21)		0.22 (0.08, 0.65)		0.38 (0.19, 0.79)		0.73 (0.29, 1.80)		0.68 (0.32, 1.47)			
**Clinical factors**												
Days with poor mental health in past 30 days (Ref. to zero day)												
At least 1 day	3.38 (2.00, 5.70)	< 0.001 ***	1.64 (0.84, 3.21)	0.148	1.46 (0.89, 2.39)	0.135	1.27 (0.70, 2.29)	0.437	1.96 (1.12, 3.44)	0.018 *	1.75 (0.89, 3.44)	0.106
Days with poor physical health in past 30 days (Ref. to zero day)												
At least 1 day	2.56 (1.59, 4.11)	< 0.001 ***	1.00 (0.51, 1.94)	0.999	2.78 (1.69, 4.58)	< 0.001 ***	2.33 (1.28, 4.22)	0.006 **	1.11 (0.66, 1.88)	0.694	NA	
Obesity (Ref. to No)												
Yes	0.81 (0.51, 1.30)	0.390	NA		1.33 (0.80, 2.22)	0.266	NA		1.31 (0.75, 2.28)	0.346	NA	
Number of chronic health conditions (Ref. to none or 1)												
> 1	4.27 (2.58, 7.07)	< .001 ***	3.52 (1.76, 7.02)	< 0.001 ***	1.84 (1.13, 2.98)	0.014 *	1.15 (0.63, 2.12)	0.645	1.55 (0.92, 2.63)	0.101	2.13 (1.11, 4.08)	0.023 *
Length of follow-up (years)												
0–24	Ref	0.005 **	HC		Ref	0.947	NA		Ref	< .001 ***	NA	
25–49	0.71 (0.43, 1.17)				1.08 (0.62, 1.86)				0.44 (0.25, 0.78)			
50 +	0.22 (0.10, 0.47)				1.10 (0.59, 2.05)				0.12 (0.04, 0.34)			

*HC*, highly correlated with one of the covariates included in the multivariable models, *NA*, not applicable covariate for not satisfying the variable selection criterium in the model fitting step

^*^p < 0.05, **p < 0.01, ***p < 0.001

#### Physical inactivity

Income was a statistically significant correlate with odds of physical inactivity. Compared to the highest income survivors (≥ $50,000), those with income between $25,000 and $35,000 (third lowest out of five income categories) had almost fourfold greater odds of physical inactivity (aOR [95%CI] = 3.85 [1.67–8.89]; data not shown in tables). Survivors reporting ≥ 1 day of poor physical health in the past 30 days, compared to those with zero day of poor physical health, had 2.3-fold odds of physical inactivity (aOR [95%CI] = 2.33 [1.28–4.22], Table 4). Age, length of follow-up from diagnosis of the primary cancer, and number of chronic conditions were not associated with odds of physical inactivity.

#### Binge drinking

As shown in Table 4, age at survey completion (≥ 65y: aOR [95%CI] = 0.15 [0.05 -0.52]; 40-64y: aOR [95%CI] = 0.42 [0.21–0.85]; reference: 18-39y), females (aOR [95%CI] = 0.30 [0.16–0.57]) and having > 1 chronic health condition (aOR [95%CI] = 2.13 [1.11–4.08]) were associated with binge drinking.

#### Multiple health-risk behaviors

Older childhood survivors had lower odds of having multiple risk behaviors (*p* = 0.003). Education (finishing college or technical school and above: aOR [95%CI] = 0.34 [0.19–0.60]; reference: high school degree or below), and multiple chronic health conditions (aOR [95%CI] = 2.33 [1.42–3.81]; reference: 0 or 1 chronic health condition) were associated with multiple health-risk behaviors (Table 5).

**Table 5:** Results from the ordinal logistic regression analysis of factors associated with the count of health-related risk behaviors among childhood cancer survivors

Characteristics	Risk behaviors (*N* = 372)			
	**Univariate analysis**		**Multivariable analysis**	
	**Odds Ratio (95% CI)**	***p*-value**	**Odds Ratio (95% CI)**	***p*-value**
**Demographic and socioeconomic factors**				
Age at survey				
18–39	Ref	< .001***	Ref	0.003**
40–64	0.66 (0.43, 1.02)		0.69 (0.43, 1.12)	
65 and older	0.30 (0.17, 0.52)		0.30 (0.15, 0.60)	
Sex: (Ref. to Male)				
Female	1.01 (0.68, 1.51)	0.953	0.68 (0.43, 1.09)	0.106
Region				
Northeast	Ref	0.403	Ref	0.830
Midwest	1.63 (0.90, 2.95)		1.11 (0.58, 2.13)	
South	1.83 (1.03, 3.26)		1.32 (0.70, 2.50)	
West	1.11 (0.65, 1.92)		1.06 (0.58, 1.92)	
Race/ ethnicity				
White only, Non-Hispanic	Ref	< .001***	Ref	0.992
Black only, Non-Hispanic	1.40 (0.52, 3.82)		1.00 (0.32, 3.14)	
Others	1.41 (0.81, 2.45)		0.96 (0.53, 1.76)	
Education Level				
High school and below	Ref	< .001***	Ref	0.001***
Attended college or technical School	0.54 (0.33, 0.89)		0.63 (0.37, 1.09)	
College or technical school and above	0.20 (0.12, 0.33)		0.34 (0.19, 0.60)	
Employment				
Employed/self-employed	Ref	< 0.001***	HC	
Out of work/A homemaker	1.21 (0.70, 2.11)			
Retired	0.37 (0.20, 0.66)			
Unable to work	2.89 (1.55, 5.39)			
Marital status				
Married/A member of an unmarried couple	Ref	0.270	NA	
Divorced/widowed/separated	1.39 (0.88, 2.19)			
Never married	1.37 (0.84, 2.24)			
Number of children in household (Ref. to No children)				
One or more	1.65 (1.10, 2.48)	0.015*	1.25 (0.78, 2.01)	0.346
Any healthcare coverage (Ref. to No)				
Yes	0.49 (0.26, 0.94)	0.032*	1.04 (0.50, 2.16)	0.917
Annual household income				
Less than $15,000	Ref	< 0.001*	Ref	0.165
$15,000 to less than $25,000	0.48 (0.23, 1.02)		0.63 (0.28, 1.40)	
$25,000 to less than $35,000	0.46 (0.20, 1.04)		0.78 (0.32, 1.94)	
$35,000 to less than $50,000	0.36 (0.16, 0.79)		0.66 (0.28, 1.60)	
$50,000 or more	0.21 (0.11, 0 .39)		0.42 (0.20, 0.89)	
**Clinical factors**				
Days with poor mental health in past 30 days (Ref. to zero day)				
At least 1 day	2.28 (1.52, 3.42)	< .001***	1.45 (0.90, 2.34)	0.123
Days with poor physical health in past 30 days (Ref. to zero day)				
At least 1 day	2.29 (1.54, 3.40)	< .001***	1.17 (0.73, 1.89)	0.517
Obesity (Ref. to No)				
Yes	1.06 (0.71, 1.58)	0.786	NA	
Number of chronic health conditions (Ref. to none or 1)				
> 1	2.72 (1.82, 4.05)	< .001***	2.33 (1.42, 3.81)	< .001***
Length of follow-up (years)				
0–24	Ref	< .001***	HC	
25–49	0.65 (0.42, 1.01)			
50 +	0.33 (0.19, 0.57)			

*HC *Highly correlated with one of the covariates included in the multivariable models, *NA *Not applicable covariate for not satisfying the variable selection criterium in the model fitting step

^*^p < 0.05, **p < 0.01, ***p<0.001

## Discussion

Childhood cancer survivors, compared to healthy controls, had higher prevalence of currently smoking (26.6% vs. 14.4%) and physical inactivity (23.7% vs. 17.7%) although both groups had similar ~ 18% prevalence of binge drinking. In our study, childhood cancer survivors had a substantial higher proportion having > 1 chronic health condition than controls and having > 1 chronic health condition was associated with higher odds of currently smoking and physical inactivity in the multivariable models. Thus, having > 1 chronic health condition could be one of the reasons why the covariate of being childhood cancer survivors (reference: controls) was not statistically significant in the multivariable models assessing factors associated with odds of currently smoking and odds of physical inactivity.

Our findings verified the double disadvantage in childhood cancer survivors, which was the aggregation of the two factors: 1) inferior health profile and 2) being more engaged with risk behaviors. We concurred with findings on the double disadvantage among adolescents with a chronic condition as Sawyer et al*.* suggests [28]. Our findings were also in congruence with the denial claim that (childhood cancer) “survivors should not be presumed to be at lower risk of engagement in risky behavior based on their vulnerable health profile” [29]. Based on our study findings, we emphasized on the importance of programs promoting healthy behaviors among childhood cancer survivors.

Robinson et al*. *reported in 2005 that the prevalence of currently smoking was 17% among 20,227 survivors from the CCSS study [8]. This prevalence was statistically lower when compared to controls who were selected as the nearest age siblings (observed to expected ratio = 0.72, 95%CI: 0.69–0.75) [8]. This prevalence was similar to findings reported from Emmons et al*.* in a 2002 study which included childhood cancer survivors of the specified age range 18-49y. In a 2012 study, using the CCSS data to compare risk behaviors between survivors and siblings (all ages 14-20y), Klosky et al*.*found the prevalence of current use of cigarette was 14.7% for survivors vs. 16.5% for siblings [29]. There have been few updated estimates of smoking prevalence among U.S. childhood cancer survivors within the last 10 years. In our study, the prevalence of currently smoking among childhood cancer survivors (26.6%) was substantially higher than the reported rates in these three previous studies. We also found higher prevalence of currently smoking in childhood cancer survivors compared to controls or siblings, which was different from results from these three previous studies [6, 8, 29] and from one of the most recent meta-analyses [30]. The difference was partially explained by the wider age range used in our analysis sample (18-80y, mean: 47y). Asfar et al*.*used national data (1997 – 2010) to report the currently smoking prevalence among adult childhood cancer survivors to be 34.6% (vs. 22.1% in controls) [31]. Our study showed the same pattern of higher prevalence of currently smoking among childhood cancer survivors when compared to controls; however, we provided the most recent estimate for this prevalence. In sum, the prevalence of currently smoking among adult childhood cancer survivors was lower than before (26.6%, reduced from 34.6%) but remained high and remained higher than controls. This updated knowledge our study provided could help timely inform public health interventions and research on risk prediction based on smoking for childhood cancer survivors.

Lower educational attainment and having > 1 chronic health condition were correlates with increased odds of currently smoking in all sample and in childhood cancer survivors alone. Smoking and chronic health conditions (including asthma, diabetes, stroke, angina or coronary heart disease) have been known to be “double harm” [32–36] while childhood cancer survivors had a substantially higher proportions of having chronic health conditions compared to controls. Among childhood cancer survivors in our study, younger survivors (< 65y) had higher odds of currently smoking, which is in agreement with the finding from a study on cancer survivors from the 2015 National Health Interview Survey [37]. Findings from our study emphasizes the importance of smoking cessation programs in childhood cancer survivors and recommends focused investment into smoking cessation programs targeting younger survivors. Higher educational attainment was associated with lower odds of physical inactivity, being obese and having > 1 chronic health condition were associated with higher odds of physical inactivity among all sample; however, such associations were not observed in the analysis on physical inactivity among childhood cancer survivors alone.

Our finding on similar rates of binge drinking between childhood cancer survivors and healthy controls concurs with previous findings from a meta-analysis [30]. However, correlates with binge drinking in the analysis of all sample and in the analysis including only childhood cancer survivors were not the same. In the analysis of all sample, reporting ≥ 1 day with poor mental health in the past 30 days was the single correlate with binge drinking from univariate analyses. Meanwhile, among childhood cancer survivors only, older ages (40-64y and > 65y; reference: 18-39y) and being female were associated with reduced odds of binge drinking and having > 1 chronic health condition was associated with increased odds of binge drinking. Our finding also concurred with Lown et al*.*study which showed that male survivors were more engaged in heavy drinking than female counterparts [38]. We suggest public health programs addressing binge drinking for childhood cancer survivors focusing on the age group of 18-39y, males, and survivors with multiple chronic health conditions.

In the assessment of the count of risk behaviors among childhood cancer survivors, we found childhood cancer survivors of older age and with higher educational attainment had lower odds of having more risk behaviors. Survivors with > 1 chronic health condition had higher odds of having more risk behaviors. Childhood cancer survivors knowingly have multiple chronic health conditions and high rates of illness [39], engagement into unhealthy risk behaviors lays severe health impacts. Consequently, programs addressing unhealthy behaviors among childhood cancer survivors with multiple chronic health conditions are priority.

Our study is not without limitations. Part of the limitations was inherent from the study design and conduct of the 2020 BRFSS. The cross-sectional design may affect measures of contemporary behaviors (including our behavior outcome of currently smoking) which may be fluctuating in the duration of the pandemic [40–42]. Meanwhile other outcomes in our study were little affected by the pandemic (i.e., physical inactivity and binge drinking were asked for lifetime experience). We believed that analyzing risk behaviors for childhood cancer survivors along with the comparison – matched healthy controls and using multivariable models for all sample including both childhood cancer survivors and controls enabled us to detect if there was any unusual estimate of currently smoking for childhood cancer survivors. Potential correlates with risk behaviors among childhood cancer survivors included type of and experience with previous treatment for the primary cancer, diet, migration background, and neurocognitive functioning [3, 23, 43, 44]; however, we cannot account for these variables since they were not available in our data. Our childhood cancer survivor sample (*N* = 372) could limit our choice of variable categorization, i.e., grouping “a student” in the same category of “out of work/a homemaker”. A quarter of our all sample lacked information on use of e-cigarette or other electronic vaping products, which impeded our capacity in conducting research on this important behavior of substance misuse. A number of correlates to health-related risk behaviors among childhood cancer survivors (e.g., past treatment for primary childhood cancer, types of childhood cancer, psychosocial information as seen in the CCSS cohort, severity of chronic health conditions) [45, 46] were not assessed in our study due to the data limitation.

Our study possessed several strengths. First, the nationally representative BRFSS offers improved generalizability on risk behaviors among U.S. childhood cancer survivors compared to previous studies [47]. Most of the previous studies on U.S. childhood cancer survivors have relied on the single large cohort of the CCSS or they collected data on a limited number of health institutions or healthcare areas [23, 48–50]. Second, our data from 2020 are timely for the development of healthcare programs aiming at promoting healthy behaviors among childhood cancer survivors; most of previous studies have come from the early 2010s or prior years [6, 7, 20, 23, 48, 49, 51]. Third, the age range of childhood cancer survivors studied in this research was not limited to survivors ≤ 49y as many previous studies have used [6, 8, 29]. Thus we were able to examine the knowledge on risk behaviors among senior childhood cancer survivors, who are the growing age sub-group of childhood cancer survivors thanks to continuing improvement in life expectancy.

In conclusion, we found significantly higher prevalence of currently smoking and physical inactivity among childhood cancer survivors and healthy controls but a similar rate of binge drinking for both groups. Every one in four childhood cancer survivors was currently smoking. Our finding indicates a possible trend of decreased smoking in this population; however, the prevalence remains high. Our study reported findings on lifestyle health-related risk behaviors for a wider age range of childhood cancer survivors compared to the previous literature. To improve their life expectancy and quality of life we recommend investment into public health programs targeting childhood cancer survivors with multiple chronic health conditions regarding their engagement in smoking and binge drinking.

### Supplementary Information

**Additional file 1:** **Figure S1.** Selection of childhood cancer survivors and controls, **Table S1.** State of residency for all participants in 2020 BRFSS.

## Data Availability

The datasets used in our study are available in the website of the U.S. Center for Disease Control and Prevention (https://www.cdc.gov/brfss/index.html).
