# Supplementary material for: Health-related risk behaviors among U.S. childhood cancer survivors: a nationwide estimate
Source: BMC Cancer. 2024 Feb 6;24:180. doi: 10.1186/s12885-024-11894-7 (PMC10845633; doi:10.1186/s12885-024-11894-7)
Supplement: Supplementary file 1 — Additional file 1: Figure S1. Selection of childhood cancer survivors and controls, Table S1. State of residency for all participants in 2020 BRFSS. [file 12885_2024_11894_MOESM1_ESM.pdf]

Health-related risk behaviors among U.S. childhood cancer survivors: A nationwide estimate

Supplemental Materials

Van T. Nghiem, PhD, MSPH,[1] Jing Jin, MS,[1] Stephen T. Mennemeyer, PhD,[1] F. Lennie Wong, PhD,[2]
[1]Department of Health Policy and Organization, University of Alabama at Birmingham School of Public Health, Birmingham, AL
[2]Department of Population Sciences, City of Hope, Duarte, CA

Figure S1: Selection of childhood cancer survivors and controls

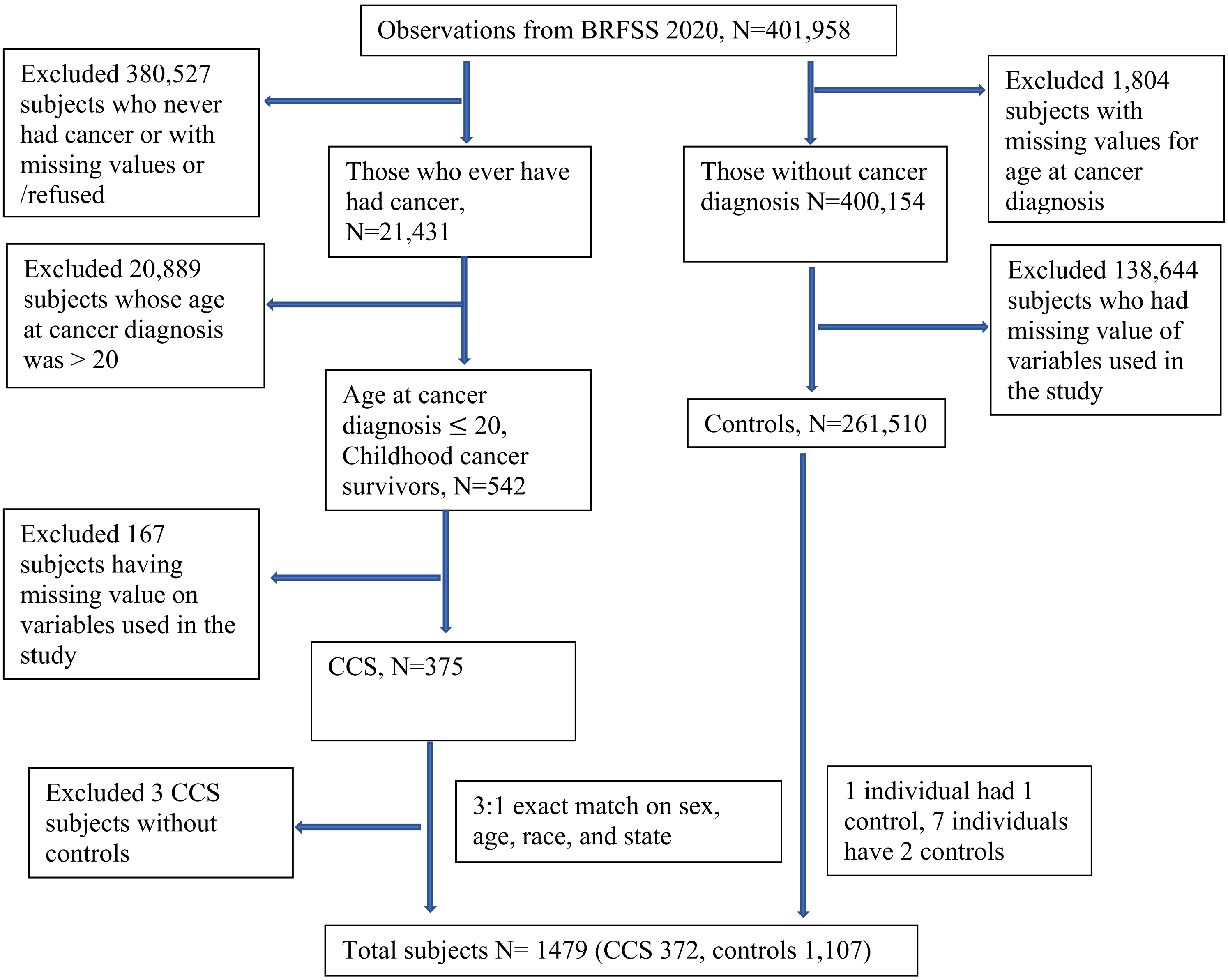

BRFSS, Behavioral Risk Factor Surveillance System, CCS, childhood cancer survivors

Table S1. State of residency for all participants in 2020 BRFSS

State	Frequency	Percent	Cumulative Frequency	Cumulative Percent
**Arizona**	88	5.95	88	5.95
**Connecticut**	88	5.95	176	11.90
**Delaware**	28	1.89	204	13.79
**Georgia**	105	7.10	309	20.89
**Hawaii**	92	6.22	401	27.11
**Indiana**	44	2.97	445	30.09
**Louisiana**	27	1.83	472	31.91
**Massachusetts**	80	5.41	552	37.32
**Michigan**	71	4.80	623	42.12
**Mississippi**	64	4.33	687	46.45
**Missouri**	108	7.30	795	53.75
**Montana**	84	5.68	879	59.43
**New Jersey**	60	4.06	939	63.49
**New Mexico**	60	4.06	999	67.55
**North Carolina**	43	2.91	1042	70.45
**Rhode Island**	60	4.06	1102	74.51
**South Dakota**	47	3.18	1149	77.69
**Utah**	163	11.02	1312	88.71
**Vermont**	47	3.18	1359	91.89
**Virginia**	80	5.41	1439	97.30
**Wisconsin**	40	2.70	1479	100.00

BRFSS, Behavioral Risk Factor Surveillance System
